# Lung transplantation in idiopathic pulmonary fibrosis: a systematic review of the literature

**DOI:** 10.1186/1471-2466-14-139

**Published:** 2014-08-16

**Authors:** Kristin D Kistler, Luba Nalysnyk, Philip Rotella, Dirk Esser

**Affiliations:** 1 Evidera, 430 Bedford Street, Suite 300, Lexington, MA 02420, USA; 2 Genzyme Corporation, Cambridge, MA, USA; 3 Boehringer Ingelheim GmbH, Ingelheim, Germany

**Keywords:** Idiopathic pulmonary fibrosis, Systematic review, Survival, Lung transplantation

## Abstract

**Background:**

Idiopathic pulmonary fibrosis (IPF) is a distinct form of interstitial pneumonia with unknown origin and poor prognosis. Current pharmacologic treatments are limited and lung transplantation is a viable option for appropriate patients. The aim of this review was to summarize lung transplantation survival in IPF patients overall, between single (SLT) vs. bilateral lung transplantation (BLT), pre- and post Lung Allocation Score (LAS), and summarize wait-list survival.

**Methods:**

A systematic review of English-language studies published in Medline or Embase between 1990 and 2013 was performed. Eligible studies were those of observational design reporting survival post-lung transplantation or while on the wait list among IPF patients.

**Results:**

Median survival post-transplantation among IPF patients is estimated at 4.5 years. From ISHLT and OPTN data, one year survival ranged from 75% - 81%; 3-year: 59% - 64%; and 5-year: 47% - 53%. Post-transplant survival is lower for IPF vs. other underlying pre-transplant diagnoses. The proportion of IPF patients receiving BLT has steadily increased over the last decade and a half. Unadjusted analyses suggest improved long-term survival for BLT vs. SLT; after adjustment for patient characteristics, the differences tend to disappear. IPF patients account for the largest proportion of patients on the wait list and while wait list time has decreased, the number of transplants for IPF patients has increased over time. OPTN data show that wait list mortality is higher for IPF patients vs. other diagnoses. The proportion of IPF patients who died while awaiting transplantation ranged from 14% to 67%. While later transplant year was associated with increased survival, no significant differences were noted pre vs. post LAS implementation; however a high LAS vs low LAS was associated with decreased one-year survival.

**Conclusions:**

IPF accounts for the largest proportion of patients awaiting lung transplants, and IPF is associated with higher wait-list and post-transplant mortality vs. other diagnoses. Improved BLT vs. SLT survival may be the result of selection bias. Survival pre- vs. post LAS appears to be similar except for IPF patients with high LAS, who have lower survival compared to pre-LAS. Data on post-transplant morbidity outcomes are sparse.

## Background

Idiopathic pulmonary fibrosis (IPF), characterized histopathologically and/or radiologically with a usual interstitial pneumonia (UIP) pattern, is the most common adult form of interstitial pneumonia of unknown origin [1]. Progressive deterioration of lung function in patients with IPF is associated with poor prognosis. Natural histories for patients with IPF vary. In most patients, the disease progresses slowly and gradually over many years. Some patients remain stable, while others have accelerated decline often associated with episodes of acute respiratory worsening or exacerbations [2-4]. The median survival time for patients with IPF is 2 to 3 years from diagnosis and the 5-year survival rate ranges between 30% and 50% [1,5-7].

There is no cure for IPF and treatment options are limited. Historically, available pharmacological therapies had limited efficacy and potential serious side effects; therefore, international guidelines concluded that there is no therapy with proven benefit to date [1]. However, just recently both nintedanib and pirfenidone have been shown to slow disease progression in separate Phase III clinical trials and for the first time, two treatment alternatives might become available for IPF patients [8,9]. Lung transplantation is so far the only treatment with proven benefit, conferring a better survival for some carefully selected patients. However, the number of lung transplantations performed is limited primarily by the supply of donor organs [10], and survival is poor for IPF patients relative to most other disease categories [11,12].

In May 2005 the United Network for Organ Sharing (UNOS) implemented the Lung Allocation Score (LAS) in the United States (US). The LAS has been utilized in Germany since December 2011, and the international exchange of donor lungs between all Eurotransplant countries is now based upon LAS. The LAS is an effort to identify the best candidates for transplant. The score is calculated using various measures of a patient’s health that estimate survival probability and projected duration of survival with or without a lung transplant. LAS scores range from 0–100 and patients with higher scores, reflecting greater predicted survival benefit, get priority. Implementation of the LAS resulted in an increased number of IPF patients receiving a lung transplant, and IPF became the most common diagnosis group to receive a lung transplant in the US in 2007 [13]. Since the implementation of the LAS system in the US, the percentage of patients on the wait list with restrictive lung disease (i.e., IPF or re-transplants) has increased from 33.8% to 46.1% [11].

Both single and bilateral lung transplantations are performed in patients with IPF, and debate remains as to whether single lung transplantation (SLT) or bilateral lung transplantation (BLT) is the better choice in this particular indication [14].

To our knowledge this is the first systematic review undertaken to summarize the published evidence on lung transplantation in IPF patients. The specific goals of this review were to summarize the published evidence on survival following lung transplantation in IPF patients, survival for SLT vs. BLT, survival post-transplant vs. wait-list survival, and survival pre- and post- LAS implementation. Data on peri-transplant complications and hospital length of stay following transplantation were summarized to the extent that data were available.

## Methods

### Literature search

A protocol detailing the methodology of this systematic review was developed. The methods used to perform this review involved both electronic and manual components, and followed established best methods used in the science of systematic review research [15-17]. A literature search was performed in MEDLINE and EMBASE to identify all English-language observational studies, published from 1990 to March 2013, reporting on lung transplantation in patients with an underlying diagnosis of IPF. Additional file 1 details the specific search strategy used. The electronic searches were supplemented by a manual search of the reference lists of all accepted studies, as well as the reference lists of recent relevant reviews. Data reported on the Organ Procurement and Transplantation Network (OPTN) and the International Society for Heart and Lung Transplantation (ISHLT) websites were also used in this review.

### Study identification

In the initial screening, abstracts were reviewed for obvious exclusion criteria, which included, guidelines, animal and *in vitro* studies, case reports, meta-analyses, clinical trials, and studies with no IPF patients or no lung transplantation. The full-text publications of accepted abstracts were reviewed to satisfy all of the pre-specified inclusion criteria. Eligible studies were those of observational design reporting on at least 10 patients with IPF who underwent lung transplantation, and reporting survival outcomes in IPF patients after lung transplantation or while on the waiting list for transplantation. Studies that only reported outcomes for a population with mixed diagnoses, such as all pulmonary fibrosis patients, without reporting IPF data separately were excluded. The most recent published reports from the ISHLT (2012 report) and the OPTN (2011 report) were included. The agreement of two investigators was required to accept or reject any articles during the review process.

### Data extraction and synthesis

Data elements of interest from each accepted study were extracted to a data extraction form. Extracted information included study-level characteristics, patient-level characteristics, and outcomes of interest. One investigator extracted the data from each study, and a second investigator independently reviewed the extracted data for completeness and accuracy.

## Results

### Study selection

**Figure 1:**
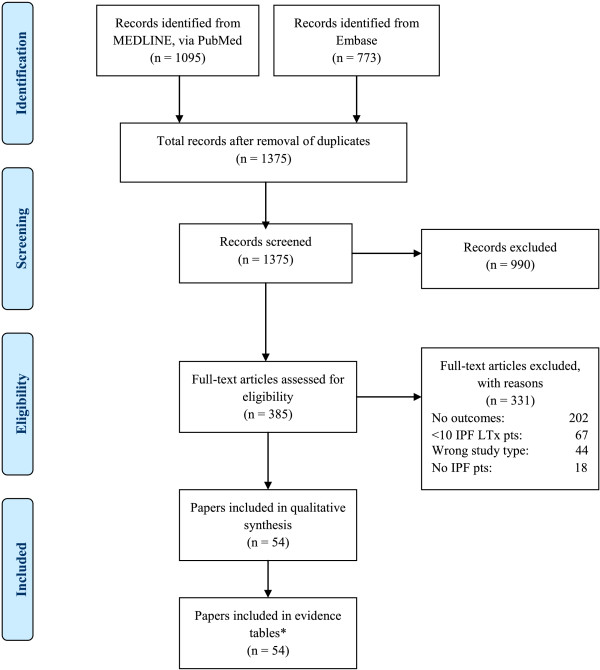
**Study attrition.** *Data from the OPTN and ISHLT websites were also included.

After removing duplicates from the various sources, the entire literature search, including manual bibliography checks, identified 1,375 citations. The majority of these citations were rejected during abstract screening, and 385 full-text articles were retrieved and screened against the protocol-specified inclusion criteria. Of these, 331 were rejected after full-text review, leaving 54 publications included in this systematic review. A flow diagram of the study attrition is presented in Figure 1.

### Study characteristics

A summary of the 56 sources (54 papers and the ISHLT and OPTN websites) is displayed in Additional file 2. The number of IPF patients in the studies ranged from 11 to 8,904. Thirteen of the 54 papers (24%) reported analyses of the OPTN data; two were analyses of the ISHLT data; six were reports of analyses from other registries or large databases; 18 papers reported data from single centers from North America; 12 papers reported data from single centers from Europe; two were from single centers from Brazil and one was a report from a single center from Australia. The mean age of the IPF patients included in the analyses was reported in 27 papers and ranged from 45.2 to 65.4 years; gender of the IPF patients was reported in 26 papers – the proportion of female patients ranged from 18.8% to 56.3%.

### Post-transplant survival

Table 1 displays a summary of post-transplant survival among IPF patients from the ISHLT and OPTN data and from the papers included in this review.

**Table 1:** Summary of post-transplantation survival among patients with IPF

**Data source**	**Yr. of transplant**	**No. of IPF patients**	**% of patients alive (95% confidence interval)**				
			**3 mo.**	**1 yr.**	**3 yr.**	**5 yr.**	**10 yr.**
***ISHLT data***							
ISHLT website & ISHLT 2012 Annual Report [12,18]	1990 – 2011	8,528	85	75	59	47	24
***OPTN data***							
OPTN website [19]	1997 – 2004^a^	1,271^b^	NR	80 (77, 83)	57 (53, 61)	41 (36, 44)	NR
2011 Annual Report [11]	2005 – 2006	755	91	81	64	53	NR
Analysis of OPTN data (*Meyer, et al*) [20]	1988 – 1997	NR	NR	NR	NR	32.1 ± 2.4% (mean±SE)	NR
Analysis of OPTN data (*Thabut, et al*) [21]	1987 – 2009	3,327	NR	76 (75, 78)	59 (57, 61)	44 (42, 46)	NR
Analysis of OPTN data (*Edwards, et al)*[22]	1996 – 2001	786	NR	~68^c^	~50^c^	~39^c^	NR
Analysis of OPTN data (*McCurry, et al*) [23]	2001 – 2006	1,636	NR	NR	NR	49	NR
Analysis of OPTN data (*Chen, et al*) [24]	2002 – 2005	1,418	NR	~79 (~75, ~82)^c^	NR	NR	NR
	2005 – 2008	1,563	NR	~80 (~78, ~83)^c^	NR	NR	NR
Analysis of OPTN data (*Freitas, et al*) [25]	1987 – 2009	4,205	NR	77^d^	58^d^	44^d^	NR
OPTN and Loyola University Medical Center databases [26]	1991 – 2009	4,190	NR	78	61	47	NR
***Other registries and large databases***							
Spanish Lung Transplant Registry (*Coll, et al*) [27]	2006 – 2010	261	~80^c^	~63^c^	NR	NR	NR
Nationwide Inpatient Sample, US (*Teo, et al*) [28]	1988 – 2006	231	In-hospital: 91	NR	NR	NR	NR
***Single center studies in Europe, Brazil and Australia***							
Single center in Denmark (*Burton, et al*) [29]	1992 – 2004	21	76	NR	52	NR	
Inova Fairfax Hospital Interstitial Lung Disease Clinic, England (*Nathan, et al*) [30,31]	2000 – 2009	84	NR	83	61	41	NR
	1996 – 2002	23	95.7	NR	NR	NR	NR
Papworth Hospital, England (*McNeil, et al*) [32]	1984 – 1994	11	46	NR	NR	NR	NR
Hopital Beaujon, France (*Thabut, et al*) [33]	1988 – 2001	28	30 day: 93	79	2 yr: 64	39	NR
University of Munich, Germany (*Neurohr, et al*) [34]	1997 – 2008	76	87	74	65	53	7.5 yrs: 40
University of Cordoba, Spain (*Algar, et al*) [35]	1993 – 2009	89	NR	53	40	33	
Santa Casa de Misericordia de Porto Alegre, Brazil (*Machuca, et al*) [36]	2004 – 2009	53	NR	72	NR	NR	NR
Alfred Hospital, Melbourne, Australia (*Keating, et al*) [37]	1990 – 2008	67	NR	76	NR	50	34
***Single center studies in North America***							
University of Pittsburgh, US (*DiGiuseppe, et al*) [38]	1986 – 2007	78		NR	NR	50	~32^c^
Two centers (Johns Hopkins Hospital and the University of Pittsburgh Medical Center), US (*Schachna, et al*) [39]	1989 – 2002	70	6-month: 80	2-year: 67	NR	NR	NR
University of Pennsylvania, US (*Rivera-Lebron, et al*) [40]	2005 – 2010	68	NR	78	NR	52	NR
Washington University School of Medicine, St Louis, Mo, US (*Meyers, et al*) [41]	1988 –1998	45	NR	76	NR	54	NR
University of California, Los Angeles, US (*Saggar, et al*) [42]	2003 – 2007	38	NR	87	NR	NR	NR
Vanderbilt University Medical Center, US (*Milstone, et al*) [43]	1990 – 1998	32	NR	25	NR	NR	NR
University of Virginia, US (*Smith, et al*) [44]	1995 – 2005	27	30 day: <60 yrs: 100; ≥60 yrs: 93 (79, 100)	<60 yrs: 85 (65, 100) ≥ 60 yrs: 93 (79, 100)	<60 yrs: 68 (42, 94) ≥ 60 yrs: 93 (79, 100)	<60 yrs: 48 (19, 78) ≥60 yrs: 58 (26, 90)	NR
University of Alabama, US (*Wille, et al*) [45]	1994 – 2004	48	87	80	67	47	NR
Washington University/Barnes Hospital (*Davis, et al*) [46]	1998 – 1993	16	In-hospital: 87	72	62	NR	NR
Toronto Lung Transplant Group, Canada (*Grossman, et al*) [47]	1983 – 1989	16	NR	63	NR	NR	NR
Cleveland Clinic, US (*Mason, et al*) [48]	1990 – 2005	82	30-day: 95	73	56	44	7-year: 36
University of Wisconsin Hospital and Clinics (*De Oliviera, et al*) [49,50]	1993 – 2009	79	In-hospital: 94	82	63	63	NR

^a^ 1-year survival based on 2002 – 2004 transplants, 3-year survival based on 1999 – 2002 transplants, 5-year survival based on 1997 – 2000 transplants.

^b^ Denominator gotten from http://optn.transplant.hrsa.gov/latestData/rptData.asp.

^c^ Percent is estimated based on graphical display of data.

^d^ Graft survival.

From the 8,528 IPF patients transplanted from 1990 to 2011 and reported to the ISHLT, the median survival was 4.5 years [12]. Survival one year post-transplantation was 75%, which decreased to 59%, 47%, and 24% at three, five, and ten years post-transplantation, respectively [12]. Post-transplant survival for IPF patients (median 4.5 years) was significantly (*P* ≤ 0.001) lower compared to patients with pre-transplant diagnoses of cystic fibrosis (CF; 7.8 years), chronic obstructive pulmonary disease (COPD; 5.4 years), or Alpha-1 - antitrypsin deficiency (6.3 years) [12]. From the 2011 OPTN Annual Report, among all US lung transplants performed in IPF patients from 2005 to 2006, survival at one year post-transplantation was 81%, which decreased to 64% at three years’ post-transplantation and 53% at five years’ post-transplantation [11]. Data available via the OPTN web site demonstrated that long-term post-transplant survival among US IPF lung transplant recipients transplanted between 1997 – 2004 was lower (3-year: 57%; 5-year: 41%) [19]. Several papers reported post-transplant survival among IPF patients from analyses of the OPTN data [20-26]; one year survival ranged from ~68% to 80%, 3-year survival ranged from 50% to 61%, and 5-year from 32% to 49%. Analyses that included earlier transplant dates tended to have lower survival than those with more recent transplant dates.

Two papers reported results from analyses of large databases other than the OPTN or ISHLT. An analysis of transplants among IPF patients from 2006 – 2010 included in the Spanish Lung Transplant Registry reported 3-month post-transplant survival of ~80% and 1-year survival of ~63% [27]. An analysis of the US Nationwide Inpatient Sample from 1988 to 2006 found that in-hospital survival among IPF patients who received lung transplants was 91% [28].

Several papers reported post-transplant survival among IPF patients from single centers across Europe and North America; one paper each reported outcomes from single centers in Brazil and Australia [29-50]. The studies included transplants performed from 1983 – 2010 and had small sample sizes (11 – 89 IPF patients). Survival at one year post-transplantation ranged from 25% to 87%; at 3 years from 40% to 67%; and at five years from 33% to 63%.

### Single vs. bilateral lung transplantation

**Figure 2:**
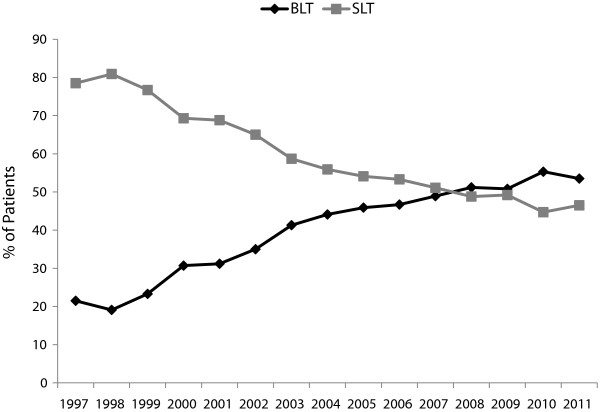
Proportion of IPF patients receiving single vs. Bilateral lung transplants from 1997 – 2011, ISHLT data.

The proportion of BLT vs. SLT procedures among IPF patients included in the ISHLT database has steadily increased since 1997 (Figure 2). In 2011, 53.5% of lung transplants among IPF patients reported to the ISHLT database were BLT and 46.5% were SLT [12,18]. In 2011, BLT accounted for 70.1% of the total number of US lung transplants (proportion of transplants among IPF patients that were BLT vs. SLT was not reported) [11]. Unadjusted analyses suggested improved long-term survival for BLT vs. SLT; (Table 2) after adjustment for patient characteristics, the differences tended to disappear.

**Table 2:** Survival following single vs. bilateral lung transplantation among IPF patients

**Data source**	**Yr. of transplant**	**No. of IPF patients**	**Survival (% IPF patients alive)**				
			**Median survival**	**1 yr.**	**3 yr.**	**5 yr.**	**10 yr.**
***ISHLT, OPTN, and Eurotransplant data***							
ISHLT data [12]	1990 - 2011	8,528	NR	SLT: 75 BLT: 74	SLT: 57 BLT: 63	SLT: 43 BLT: 49	SLT: 20 BLT: 35
Analysis of OPTN data (*Force, et a*l) [51]	1987 - 2008	3,830	NR	NR	NR	SLT ~53^a^	SLT ~30^a;^
						BLT: 60^a^	BLT: ~48^a^
Analysis of OPTN data (*Thabut et al*) [21]	1987 - 2009	3,327	SLT: 5.2 years (4.3–6.7); BLT: 3.8 years (3.6–4.1)	NR	NR	NR	NR
Analysis of OPTN data (*Nwakanma, et al*) [52]	1998 - 2004	429	30-day SLT: 94	SLT: 69	SLT: 52	SLT: 33	NR
			BLT: 95	BLT: 72	BLT: 54	BLT: 54	
Eurotransplant (*Smits et al*) [53]	1997 - 1999	104	NR	NR	SLT: 63	NR	NR
					BLT: 47		
***Single center studies***							
University of Wisconsin Hospital and Clinics (*De Oliveira, et al*) [49,50]	1993 - 2009	79	NR	SLT: 82	SLT: 65	SLT: 65	SLT: 49
				BLT: 86	BLT: 55	BLT: 55	BLT: 55
Cleveland Clinic, US (*Mason, et al*) [48]	1990 - 2005	82	NR	SLT: 67	NR	SLT: 34	NR
				BLT: 81		BLT: 55	
Alfred Hospital, Melbourne, Australia (*Keating, et al*) [37]	1990 - 2008	67	NR	SLT: 78	NR	SLT: 49	SLT: 29
				BLT: 68		BLT: 50	BLT: 50
University of Munich, Germany (*Neurohr, et al*) [34]	1997 - 2008	76	SLT: 83	SLT: 70	SLT: 55	SLT: 42	NR
			BLT: 93	BLT: 80	BLT: 74	BLT: 67	

^a^Percent is estimated based on graphical display of data.

For lung transplants received by IPF patients from 1990 to June 2011 and reported to the ISHLT, survival was significantly (P < 0.001) better for BLT vs. SLT [12]. At one year post-transplant, the proportion of IPF patients alive was similar between BLT (73.9%) and SLT (74.8%); long-term follow-up showed that a greater proportion of IPF patients who received BLT vs. SLT were alive (3-year post-transplant, BLT: 63.0%; SLT: 56.7%; 5-year: BLT: 49.4%; SLT: 43.3%; 10-year: BLT: 34.9%; SLT: 20.0%) [12].

Data from the OPTN also suggest improved survival for BLT vs. SLT. Among 3,830 IPF patients included in an analysis of the OPTN data who received transplants between 1997 and 2008, patients who received a BLT had significantly (*P* < 0.001) longer mean survival (8.34 years) compared to patients who received a SLT (7.37 years), (HR for mortality: 0.798 [95% CI 0.716 to 0.889]) [51]. Long-term survival, conditional to surviving one-year, was also significantly (*P* < 0.0006) better for patients who received a BLT vs. SLT (12.08 years versus 6.8 years) [51]. At five years’ post-transplantation, approximately 60% and 53% of patients who received BLT and SLT transplants, respectively, were alive [51]. At ten years’ post-transplantation, approximately 48% and 30% of patients who received BLT and SLT transplants, respectively, were alive [51]. An analysis of the OPTN data that included 92 (27 BLT) adults with IPF transplanted from 1987 to 1997 who survived ≥10 years after their first lung transplant and 205 (30 BLT) adults with IPF who received an allograft within the same era (1987–1997) and died between 1 and 5 years after lung transplantation, found that longer post-transplant survival among IPF patients was associated with BLT – 29.4% of 10-year survivors underwent BLT compared to only 14.6% of 1–5-year survivors (*P* = 0.003) [54]. Another analysis of the OPTN data that included 3,327 IPF patients transplanted between 1997 and 2009 also found significantly (*P* < 0.001) longer median survival after BLT (5.2 years [95% CI, 4.3 to 6.7 years]) vs. SLT (3.8 years [95% CI, 3.6 to 4.1 years]) [21]. However, the difference was not evident in an analysis restricted to 1,218 patients who had lung transplantation from 2002 onward (BLT: 5.0 years vs SLT: 4.6 years, *P* = 0.29). An unadjusted analysis suggested lower mortality with BLT vs. SLT (HR: 0.80 [95% CI, 0.71 to 0.89]), but the effect was no longer statistically significant after adjustment for recipient, donor, and procedure-related variables (HR: 0.92 [95% CI, 0.81 to 1.06]) [21]. The authors conducted further analyses and concluded that there was possibly an increased relative risk for death with BLT in the early postoperative period, followed by a decreased relative risk for death long-term [21]. A single center study from the US of 82 IPF patients who received a lung transplant found that one and five year survival was significantly greater following BLT vs. SLT [48]. However, when BLT vs. SLT survival was assessed using IPF patients matched on potential risk factors, there was no significant difference between transplantation types (*P* = 0.3). The matched analysis was limited by a very small sample of 10 BLT and 10 SLT patients. The authors suggested that SLT was a marker for a high-risk subgroup of older patients with IPF, rather than being a true risk factor for increased mortality [48].

Other analyses, including 1994 – 2004 data from the ISHLT [55], OPTN data [52], a single center study from the US [50], and a single center study from Australia [37], found no statistically significant difference in the survival between IPF patients receiving BLT and receiving SLT. However, survival tended to be numerically greater for BLT vs. SLT.

In contrast, an analysis of the Eurotransplant data which includes data from Austria, Belgium, Germany, The Netherlands, Luxembourg and Slovenia, found that 3-year survival among 104 transplants received between 1997 – 1999, was significantly (*P* = 0.04) better for SLT (63%) compared to BLT (47%) in IPF patients [53].

Two papers reported data on post-transplant in-patient mortality. From an analysis of the US National Inpatient Sample database, in-hospital death was significantly (*P* = 0.006) greater following SLT (16%) vs. BLT (5%) [28]. From a small single center study from the US that included 45 IPF patients, there was no significant difference in in-hospital mortality between BLT (9.4%) and SLT (9.4%) [41].

### Post-transplant survival vs. wait list survival

In 2011, patients with IPF accounted for the largest proportion (46.1%) of patients on the lung transplant wait-list in the US [19]. The median time on wait list to transplant for IPF patients in the US has decreased from 4.1 months in 2005 to 2.1 months in 2011 [19]. Correspondingly, the percent of adults with IPF on the wait list who received a lung transplant within a year increased from 27% in 1998 to 71.2% in 2010 [19]. Despite the fact that the median wait-list time to transplant was lower for IPF vs. other diagnosis categories and that the transplant rate was highest among IPF patients (165.9/100 person-years of waiting time vs. 119.5, 46.9, and 66.3 for patients with obstructive lung disease, pulmonary vascular disease, and CF, respectively), OPTN data show that the pre-transplant mortality rate among adult IPF patients wait-listed for a lung transplant (26.9 / 100 patient years of waiting time) was higher for IPF vs. other underlying causes of disease (Table 3) [19]. An analysis of 61 IPF patients awaiting lung transplantation in five institutions and listed in The Korean Network for Organ Sharing (KONOS) from May 1996 to May 2011, found that mean survival time for IPF patients who died on the waiting list was 7.9 ± 15.5 months [56]. An analysis of 24 IPF patients referred for lung transplantation and lung-and-heart transplantation at a single center in Poland between September 1999 and December 2004 found that the mean time to death was 11.6 ± 10.5 months [57].

**Table 3:** Pre-transplant mortality rates among adult patients wait-listed for a lung transplant

	**Obstructive lung disease**^**1**^	**Pulmonary vascular disease**^**2**^	**Cystic fibrosis and immunodeficiency disorders**	**Idiopathic pulmonary fibrosis and re-transplant**
1998-99	10.8	17.2	21.6	32.1
2000-01	9.7	13.1	17.8	23.1
2002-03	8.7	10.6	14.8	22.1
2004-05	7.7	7.3	14.4	19.9
2006-07	6.9	8.7	10.7	19.2
2008-09	5.5	14.2	15.3	22.5
2010-11	6.7	19.9	15.5	26.9

^1^e.g., chronic obstructive pulmonary disease/emphysema.

^2^e.g., idiopathic pulmonary arterial hypertension.

Mortality rates are computed as the number of deaths per 100 patient years of waiting time in the given 2-year interval. Waiting time is calculated as the total waiting time in the interval for patients in that group. Only deaths that occur prior to removal from the waiting list are counted.

From the 13 papers that reported data on wait list mortality in IPF patients, the proportion of IPF patients who died while awaiting transplantation ranged from 14% to 67% (Table 4). An analysis of 53 IPF patients enrolled at a lung transplant program in Italy and assessed over a 100 month study period, found that mortality rate before lung transplantation for IPF was higher than after lung transplantation (44% vs 16%) [58]. An analysis that included 134 patients in the UK and Ireland found that, of those who died while awaiting lung transplantation, IPF was the leading cause of death. Among all lung transplant recipients and wait-list patients (regardless of underlying diagnosis), survival was significantly (*P* < 0.001) better among those who received a transplant [59]. From a single center study in France that included 46 IPF patients on the waiting list from 1988 to July 2001, lung transplantation in patients with IPF was associated with a 75% reduction in the risk of death (HR: 0.25 [95% CI: 0.08-0.86] *P* = 0.03) [33].

**Table 4:** Summary of wait list mortality in IPF patients

	**No. of IPF patients**	**% died on wait list**
OPTN data, IPF patients on the lung transplant list between January 1995 and December 2000 [60]	2,115	31%
IPF patients, recorded in the OPTN database between June 30, 2004 and July 22, 2005, with 6-months of follow-up who did not undergo lung transplantation [61]	209	23%
IPF patients on the wait list at the University of California San Diego from January 1990 to February 1999 [62]	25	28%
IPF patients awaiting lung transplantation in five institutions and listed in The Korean Network for Organ Sharing (KONOS) from May 1996 to May 2011 [56]	61	57%
IPF patients who underwent assessment for lung transplantation at a single center in the UK [63]	42	64%
IPF patients at Inova Fairfax Hospital who were on the wait list for lung transplantation from 2000 to 2005 [64]	74	18%
IPF patients assessed for lung transplantation from January 1991 to June 1995 at the Toronto Lung Transplant Program [65]	26	19%
single center study of IPF patients at a single institution in Brazil registered on the waiting list from 2001 to June 2008 [66]	33	24%
single center study in France that included 46 IPF patients on the waiting list from 1988 to July 2001 [33]	46	35%
IPF patients referred for lung transplantation and lung-and-heart transplantation at a single center in Poland September 1999 and December 2004 [57]	24	67%
IPF patients enrolled at a lung transplant program in Italy (100 month time period) [58]	53	44%
Consecutive IPF patients assessed for lung transplantation between January 1997 and May 2006 at a single center in Israel (Pulmonary Institute of Rabin Medical Center) [67]	85	48%
IPF Patients enrolled from January – June 2004 at a single center in Israel (Pulmonary Institute of Rabin Medical Center) and followed from enrollment for a median of 2.4 years (range 2.0 to 3.1 years) [68].	51	14%

### Cause of death among IPF patients

Among IPF patients who received a lung transplant in the US from 1987 to 2009, the leading cause of death was infection (24% of deaths) [21]; papers from single center studies and one dual center study, also reported that infection or sepsis was a leading cause of death [33,34,39,41,42,45,48,69], along with bronchiolitis obliterans syndrome (BOS), chronic rejection [33,34,41,42,45,47], or unspecified graft failure [70], (Additional file 3).

### Burden post-transplantation

Few papers reported information on post-transplant burden. The papers that did report post-transplant hospital length of stay and adjunct interventions suggest that lung transplantation in IPF patients is associated with substantial resource use (Additional file 4).

### Transplant era and LAS score

From the ISHLT data, a multivariable analysis of 4,463 IPF patients who received lung transplants from 1999 – 2011 found that patients transplanted from 2010 to 2011 had a significantly decreased 1-year mortality risk compared to patients transplanted between 2005 – 2006 (2005/2006 vs. 2010/2011: HR: 1.46 [95% CI: 1.2 – 1.8] *P* = 0.0013); 2003 – 2004 (HR: 1.70 [95% CI: 1.3 – 2.2] *P* < 0.001); 2001 – 2002 (HR: 1.99 [95% CI: 1.5 – 2.7] *P* < 0.001); and 1999 – 2000 (HR: 2.98 [95% CI: 2.2 – 4.0] *P* < 0.0001) [12]. Patients transplanted from 2007 – 2008 had a borderline increased 1-year mortality risk compared to patients transplanted from 2010 – 2011 (HR: 1.24 [95% CI 1.0 – 1.5] *P* = 0.0525) [12].

An analysis of the OPTN data which compared patients transplanted post-LAS (May 1, 2005 until April 30, 2008) to those transplanted pre-LAS (i.e., from May 1, 2002 through April 30, 2005) found that, among all lung transplant patients regardless of underlying diagnosis, survival at one year post-transplantation was similar between the pre- and post-LAS periods [71]. A single center study from the US found no significant (*P* = 0.98) difference in long-term survival for IPF patients transplanted pre-LAS (n = 33) vs. post-LAS (n = 46) [49]. For the pre-LAS group, actuarial survival at 1 and 3 years were 78.8% and 63.6%, respectively. For the post-LAS group, actuarial survival at 1 and 3 years were 85.8% and 62.8%, respectively.

From an analysis of the OPTN data, IPF patients with high LAS (>46) had significantly (*P* = 0.01) decreased one year survival (76.4%) compared to IPF patients with a low LAS (≤46) (82.6%) [71]. Compared to IPF patients in the pre-LAS group, IPF patients with LAS ≤46 did not have elevated risk of death (HR: 0.98 [95% CI 0.79–1.22]), though IPF patients with LAS >46 did (HR: 1.39 [95% CI: 1.07–1.81]) [71]. A single center study from the US also found that IPF patients with a high LAS (≥50) had significantly (*P* = 0.013) poorer one year survival (65.1%) compared to IPF patients with a low-LAS (<50) (79.9%) [72].

## Discussion

Lung transplantation is now a well-accepted treatment option for the management of a wide range of chronic end-stage lung disorders, including IPF. The primary goal of lung transplantation is to provide a survival benefit for patients who are failing medical therapy or for whom there is no effective medical treatment. However, there are far fewer available donor organs than patients that would potentially benefit from the lung transplantation procedure. In 2011, approximately 886 IPF patients in the US underwent lung transplantation [19]. Using a prevalence of 14–43 per 100,000 [73] and a US adult population of approximately 311 million in 2011 [74], less than 1% of the estimated 42,000–130,000 US IPF patients had received a lung transplant during 2011. Not all IPF patients would be eligible for or willing to undergo transplantation, thus this probably overestimates the proportion not receiving a transplant. Nonetheless, there remains a large gap between those eligible and those receiving a transplant. Therefore, it is important that the available resources are optimally used, so that the patients selected for transplantation have the best chances for favorable long-term outcomes. There is no cure for IPF and current guidelines for the selection of lung transplant candidates recommend that appropriate IPF patients should be referred for transplantation as early as possible [75].

In this review we summarized lung transplantation outcomes in IPF patients using recent evidence. The published data and publically available data from the OPTN and ISHLT demonstrate that approximately 50% of IPF patients are alive at five years’ post-transplantation. IPF patients continue to have poorer survival compared to lung transplant patients with other underlying diagnoses. IPF patients also have a higher wait-list mortality compared to other diagnoses. The high proportion of IPF patients dying prior to receipt of a lung transplant and the low survival time among those who do die, support the benefits of lung transplantation as a treatment for IPF patients as well as the high unmet need in this patient population. However, there is the potential for a survivor treatment selection bias in the comparison of wait list vs. post-transplant survival — IPF patients who receive lung transplants have to live long enough to receive a donor lung, so if they are compared to the non-transplant group they may appear to have better survival. There has been a significant increase in the proportion of IPF patients receiving BLT vs. SLT. Data tend to suggest that BLT is associated with improved long-term survival compared to SLT. However, the apparent improved long-term survival may be due to the SLT patients being at higher risk for poor survival rather than effects of the BLT per se. Given the shortage of donor lungs available, it is important to understand whether there is a subgroup of IPF patients for whom BLT does offer a true survival advantage and whether it would be beneficial to reserve bilateral transplants for those patients. Data specifically comparing survival among IPF patients who received a lung transplant pre- vs. post-LAS are limited. However, implementation of the LAS has resulted in an increase in lung transplants due to IPF, and has resulted in shorter wait times when compared to patients in the pre-LAS period (due to patients being listed later since wait time is no longer a primary factor in determining allocation, and due to the fact that IPF patients are selected for transplants based on their LAS). The ISHLT data show that longer 1 year survival is associated with more recent transplant year; this could be an effect of the LAS system but may also be due to other factors such as improvements in medical care and immunosuppression drug regimens. Other than mortality data, there are few reported data on post- or peri-transplant outcomes among IPF patients. The data reported in the papers included in this review suggest that transplantation for IPF is associated with considerable resource allocation beyond the actual transplantation procedure; this includes substantial ICU length of stay as well as necessity for adjunct interventions such as tracheostomy and use of inhaled nitric oxide and prolonged mechanical ventilation.

To our knowledge, this is the first systematic and most extensive review of the available evidence on lung transplantation outcomes in IPF patients, evaluating the published literature over more than two decades. The systematic methodology employed for this effort and the extensive nature of the outcomes assessed (i.e., wait list and post-transplant survival; pre- vs. post-LAS survival; BLT vs. SLT survival; post-transplant morbidity) are strengths of this review. A substantial amount of the reviewed evidence was derived from OPTN or ISHLT data. While the availability if the OPTN and ISHLT data contributes extensively to the understanding of post-transplant survival, detailed post-transplant outcomes are not collected. A limitation of the data summarized in this review is the lack of actual confirmation of IPF in most of the studies.

## Conclusions

This review highlights that data on post-transplant morbidity and resource use outcomes among IPF patients compared to other lung transplant patients are lacking. In addition, a greater understanding of which IPF patients should receive BLT is needed. While lung transplant appears to offer a survival advantage when compared to wait list IPF patients, IPF patients still have the lowest survival of all lung transplant patients; this coupled with the lack of available donor lungs, suggests a high need for effective medical therapy.

## Abbreviations

BLT: Bilateral lung transplantation; BOS: Bronchiolitis obliterans syndrome; CF: Cystic fibrosis; COPD: Chronic obstructive pulmonary disease; HR: Hazard ratio; ICU: Intensive care unit; IPF: Idiopathic pulmonary fibrosis; ISHLT: International Society for Heart and Lung Transplantation; KONOS: Korean Network for Organ Sharing; LAS: Lung Allocation Score; OPTN: Organ Procurement and Transplantation Network; SLT: Single lung transplantation; UIP: Usual interstitial pneumonia; UK: United Kingdom; UNOS: United Network for Organ Sharing; US: United States.

## Competing interests

D.E. is an employee of Boehringer Ingelheim GmbH, funding for this study was provided to Evidera by Boehringer Ingelheim GmbH.

P.R. and K.K. are employees of Evidera, funding for this study was provided to Evidera by Boehringer Ingelheim GmbH.

L.N. was an employee of Evidera (formerly United Biosource) at the time of the study.

## Authors’ contributions

LN, PR, DE, made substantial contributions to conception and design; PR and KK carried out the literature review; all authors contributed to the interpretation of data; LN, PR, and KK were involved in drafting the manuscript; DE and KK were involved in revising the manuscript critically for important intellectual content; all authors read and approved the final manuscript.

## Authors’ information

Note: Luba Nalysnyk was employed by United BioSource Corporation at the time of the review.

## Pre-publication history

The pre-publication history for this paper can be accessed here:

http://www.biomedcentral.com/1471-2466/14/139/prepub

## Supplementary Material

Additional file 1 Search Strategy to Identify Papers Indexed in MEDLINE and EMBASE and Reporting Data on Post-Transplantation Survival Among Patients with IPF. Click here for file

Additional file 2 Summary of the 56 sources included in the review. Click here for file

Additional file 3 Summary of cause of death in IPF patients. Click here for file

Additional file 4 Post-transplant length of stay and adjunct interventions in IPF patients. Click here for file
