# Supplementary material for: Lung transplantation in idiopathic pulmonary fibrosis: a systematic review of the literature
Source: BMC Pulm Med. 2014 Aug 16;14:139. doi: 10.1186/1471-2466-14-139 (PMC4151866; doi:10.1186/1471-2466-14-139)
Supplement: Additional file 1 — Search Strategy to Identify Papers Indexed in MEDLINE and EMBASE and Reporting Data on Post-Transplantation Survival Among Patients with IPF. [file 1471-2466-14-139-S1.doc]

Additional File 1. Search Strategy to Identify Papers Indexed in MEDLINE and EMBASE and Reporting Data on Post-Transplantation Survival Among Patients with IPF

| MEDLINE Search (via PubMed)  1. “lung transplantation”[MeSH] OR lung transplant* OR lung graft 2. “idiopathic pulmonary fibrosis”[MeSH] OR “IPF”[tiab] OR “pulmonary fibrosis”[MeSH] OR “pulmonary fibrosis” OR “lung diseases, interstitial”[MeSH] OR interstitial lung disease* OR “ILD”[tiab] OR “fibrosing alveolitis” OR “CFA”[tiab] OR “usual interstitial pneumonia” OR “UIP”[tiab] OR idiopathic interstitial pneumonia* OR “IIP”[tiab] 3. “animals”[MeSH] NOT “humans”[MeSH] 4. (#1 AND #2) NOT #3   Limits: English, Published from 1990/01/01 to 2013/03/28 |
| --- |
| Embase Search  1. ‘lung transplant’ OR ‘lung transplants’ OR ‘lung transplantation’ OR ‘lung transplantations’ OR ‘lung graft’ OR ‘lung grafts’ OR ‘lung grafting’ OR ‘lung graftings’ 2. ‘fibrosing alveolitis’/exp OR ‘fibrosing alveolitis’ OR ‘pulmonary fibrosis’ OR ‘interstitial lung disease’ or ‘interstitial lung diseases’ OR ‘usual interstitial pneumonia’ OR ‘idiopathic interstitial pneumonia’ or ‘idiopathic interstitial pneumonias’ 3. [animals]/lim NOT [humans]/lim 4. [cochrane review]/lim OR [systematic review]/lim OR [conference paper]/lim OR [conference abstract]/lim OR [editorial]/lim OR [erratum]/lim OR [note]/lim OR [review]/lim 5. (#1 AND #2) NOT (#3 OR #4)   Limits: English, Published from 1990/01/01 to 2013/03/28 |
