# Supplementary material for: Lung transplantation in idiopathic pulmonary fibrosis: a systematic review of the literature
Source: BMC Pulm Med. 2014 Aug 16;14:139. doi: 10.1186/1471-2466-14-139 (PMC4151866; doi:10.1186/1471-2466-14-139)
Supplement: Additional file 2 — Summary of the 56 sources included in the review. [file 1471-2466-14-139-S2.doc]

**Additional File 2. Summary of the 56 sources included** in the review

| **Reference** | **Sample source** | **No. of IPF patients** | **Mean + SD age** | **% females** |
| --- | --- | --- | --- | --- |
| ISHLT data | | | | |
| ISHLT website[12] | ISHLT data (website)1995 – 2012 | 8,904 | NR | NR |
| Christie, et al[18] | ISHLT data (2012 annual report), 1995 – 2011 | 7,925 | NR | NR |
| Trulock, et al[55] | ISHLT data, All adult IPF lung transplant recipients, 1994–2004 | 2,318 | NR | NR |
| OPTN data | | | | |
| OPTN website[19] | OPTN data, All adult IPF lung transplant recipients, 1997 – 2004 | 1,271 | NR | NR |
| McCurry, et al[23] | OPTN data, All IPF lung transplant recipients, 2007 | NR | NR | NR |
| Edwards, et al[22] | OPTN data, Adult IPF lung transplant recipients 1987–2002 | 786 | NR | NR |
| Force, et al[51] | OPTN data, All IPF lung transplant recipients, 1987–2008 | 3,860 | SLT: 56.5 + 9.5 BLT: 51.8 + 12.2 | 39.7 |
| Freitas, et al[25] | OPTN data, All IPF lung transplant recipients, 1987–2010 | 4,205 | NR | NR |
| Merlo, et al[69] | OPTN data, All IPF lung transplant recipients, 2002 - 2008 | 1,241 | NR | NR |
| Meyer et al[20] | OPTN data, All IPF lung transplant recipients, 1988 - 1999 | NR | NR | NR |
| Nwakanma, et al[52] | OPTN data, All IPF lung transplant recipients, 1998 - 2004 | 429 | NR | NR |
| Takagishi, et al[26] | OPTN data, All IPF lung transplant recipients, 1991 - 2009 | 4,190 | 65.4 ± 10.1 | 31.9 |
| Thabut, et al[21] | OPTN data, Adult IPF lung transplant recipients, 1987 - 2009 | 3,327 | SLT: 57.1 + 9.0 BLT: 54.0 + 10.0 | 32.0 |
| Weiss, et al[54] | OPTN data, Adult IPF lung transplant recipients, 1987 – 1997 | NR | NR | NR |
| Chen, et al[24] | OPTN data, IPF lung transplant recipients, 2002 - 2008 | NR | NR | NR |
| Lederer, et al[61] | OPTN data, IPF patients listed for lung transplant, 2004 - 2005 | 454 | 56 ± 9 | 33 |
| Shorr, et al[60] | OPTN data, All IPF patients on the waiting list for lung transplantation, 1995 - 2000 | 2,115 | 51.4 ± 9.6 | 39.3 |
| Other registry data or large databases | | | | |
| Coll, et al[27] | IPF lung transplant recipients, Spanish Lung Transplant Registry, 2006 - 2010 | 261 | NR | NR |
| Smits, et al[53] | Adult IPF lung transplant recipients, Eurotransplant database, 1997-1999 | 104 | NR | NR |
| Grossman, et al[47] | IPF single-lung transplant recipients, Toronto Lung Transplant Group, 1983 - 1989 | 16 | 51.4 ± 7.7 | 18.8 |
| Kadikar, et al[65] | IPF patients evaluated for lung transplantation, Toronto Lung Transplant Program, 1991–1995 | 26 | 52.1 ± 6.0 | 23.1 |
| Teo, et al[28] | Adults with IPF (ICD-9 code) as primary inpatient diagnosis and inpatient lung transplantation codes, Nationwide Inpatient Sample database, 1988–2006 | 231 | NR | NR |
| Paik, et al[56] | All IPF patients on the lung transplant waiting list, KONOS registry, 1996–2011 | 61 | NR | NR |
| Single center data, North America | | | | |
| Davis, et al[46] | All adult IPF single-lung transplant recipients, Washington University/Barnes Hospital 1988 - 1993 | 16 | 45.2 ± 10.5 | 56.3 |
| Meyers et al, [41] | All IPF lung transplant recipients, Washington University/Barnes Hospital, 1988–1998 | 45 | 49.5 ± 10 | 42.2 |
| De Oliveira, et al[49] | Consecutive IPF lung transplant recipients, University of Wisconsin Hospital and Clinics, 1993 - 2009 | 79 | Pre-LAS: 52.5 ± 9.9 Post-LAS: 57.9 ± 6.5 | 19 |
| Di Giuseppe, et al [38] | All IPF lung transplant recipients, University of Pittsburgh, 1986 - 2007 | 79 | 54.5 ± 10.3 | 32.9 |
| Mason, et al[48] | All IPF lung transplant recipients, Cleveland Clinic, 1990 - 2005 | 82 | 52 ± 11 | 36.6 |
| Milstone et al [43] | IPF lung transplant recipients, Vanderbilt University Medical Center, 1990–1998 | 32 | CMV-: 51 + 6 CMV+ 54 + 9 | 28.1 |
| Nathan, et al[31] | IPF lung transplant recipients, Inova Fairfax Hospital, 1996–2002 | 23 | Patients with PE event: 58.7 + 4.3 No PE event: 54.7 + 7.0 | 39.1 |
| Nathan, et al[30] | IPF patients evaluated for lung transplantation, Inova Fairfax Hospital,2000 - 2009 | 521 | 65 + NR | 28.3 |
| Nathan, et al[64] | IPF patients listed for lung transplantation, Inova Fairfax Hospital, US, 2000–2005 | 73 | NR | NR |
| Rivera-Lebron, et al[40] | IPF patients evaluated for lung transplant with ECG and RHC reported, Hospital of the University of Pennsylvania, 2005–2010 | 135 | 58 ± 7 | 27 |
| Saggar, et al[42] | All IPF bilateral lung transplant recipients, University of California, Los Angeles, 2003–2007 | 38 | 58.8 + NR | 23.7 |
| Schachna, et al[39] | All IPF lung transplant recipients, Johns Hopkins Hospital; University of Pittsburgh Medical Center, 1989–2002 | 70 | 55.7 ± 8.6 | 40.0 |
| Smith, et al[44] | Adult IPF lung transplant recipients, University of Virginia, 1995–2005 | 27 | NR | NR |
| Wille, et al[45] | All IPF lung transplant recipients, University of Alabama at Birmingham, 1994–2004 | 48 | 55.8 ± 7.9 | 41.7 |
| De Oliveira, et al[50] | Consecutive IPF patients who underwent lung transplantation, University of Wisconsin Hospital and Clinics, 1993–2009 | 79 | SLT: 56.8 ± 7.2 BLT: 43.4 ± 12.3 | 19 |
| Horai, et al[70] | Consecutive IPF lung transplant patients, University of Pittsburgh Medical Center, 2005–2010 | 140 | NR | NR |
| Timmer, et al[62] | Adult lung transplant candidates with a pathological diagnosis of UIP, University of California San Diego, 1990–1999 | 25 | NR | NR |
| Erasmus, et al[70] | IPF lung transplant recipients with large airway complications, Mayo Clinic Jacksonville, US, 2001–2007 | 55 | NR | NR |
| Single center data, Europe | | | | |
| McNeil, et al [32] | IPF single-lung transplant recipients, Papworth Hospital, UK, 1984–1994 | 11 | NR | NR |
| Mackay, et al[63] | All IPF lung transplant recipients, Freeman Hospital, UK, 1999–2004 | 17 | 53.8 + NR | NR |
| Neurohr, et al[34] | Consecutive IPF lung transplant recipients, Ludwig-Maximilians University of Munich, 1997–2008 | 76 | SLT: 53.74 ± 1.15* BLT: 50.38 ± 1.34* | 43.4 |
| Thabut, et al[33] | IPF lung transplant recipients, Beaujon Hospital, Clichy, France, 1988–2001 | 28 | 49.3 + NR | 28.6 |
| Algar, et al[35] | IPF lung transplant recipients, University of Cordoba, Spain, 1993–2009 | 89 | 51 ± 11 | 28.1 |
| Burton, et al[29] | All IPF patients receiving deceased donor lung transplants, Rigshospitalet, Copenhagen University Hospital, Denmark, 1992–2004 | 21 | NR | NR |
| Callegari, et al[58] | IPF patients enrolled at a lung transplant program, Pulmonary Division Medical Centre of Montescano, Italy, 1991–1999 | 53 | NR | NR |
| Jastrzebski, et al[57] | Adult IPF lung transplant candidates, Silesian School of Medicine, Zabrze, Poland, 1999–2005 | 24 | NR | NR |
| Shitrit, et al[67] | Consecutive IPF patients registered on the waiting list for lung transplantation, Rabin Medical Center, Israel, 1997–2006 | 85 | NR | NR |
| Shitrit, et al[68] | IPF patients with progressive symptomatic and/or physiologic deterioration despite treatment with prednisone ± immunosuppresive agents, Rabin Medical Center, Israel, 2004 | 51 | 58 ± 11 | 43.1 |
| Rusanov, et al[69] | IPF patients with progressive symptomatic and/or physiologic deterioration despite treatment with prednisone ± immunosuppresive agents, Rabin Medical Center, Israel, 2009 | 61 | 61 ± 7 | 32.8 |
| Adamali, et al[59] | All Irish citizens with IPF who underwent lung transplantation at MMUH, Freeman Hospital in Newcastle, Harefield Hospital in Middlesex, or Great Ormond Street in London, 2000–2011 | 20 | 56.3 ± 1.4 | 35.0 |
| Single center data, South America, Australia | | | | |
| Costa da Silva, et al[66] | IPF patients at a single institution in Brazil registered on the waiting list from 2001 to June 2008 | 33 | NR | NR |
| Machuca, et al [36] | All IPF lung transplant recipients, Santa Casa de Misericordia de Porto Alegre, Brazil, 2004 - 2009 | 53 | NR | NR |
| Keating, et al[37] | IPF lung transplant recipients, Alfred Hospital, Melbourne, 1990 - 2008 | 67 | NR | NR |

MMUH: Mater Misericordiae University Hospital [Dublin, Ireland]; PE: Pulmonary Embolism; UIP: usual interstitial pneumonitis; * standard error
