# Supplementary material for: Lung transplantation in idiopathic pulmonary fibrosis: a systematic review of the literature
Source: BMC Pulm Med. 2014 Aug 16;14:139. doi: 10.1186/1471-2466-14-139 (PMC4151866; doi:10.1186/1471-2466-14-139)
Supplement: Additional file 3 — Summary of cause of death in IPF patients. [file 1471-2466-14-139-S3.doc]

**Additional File 3. Summary of cause of death in IPF patients**

| **Source** | **Yr. of transplant** | **No. of patients** | **No. of deaths** | **Cause of death** | | |
| --- | --- | --- | --- | --- | --- | --- |
| ***OPTN Data*** | | | | | | |
| Analysis of OPTN data (*Thabut et al*) [21] | 1987 - 2009 | 3,327 | 1,556 | Infection: 23.6%  Primary graft failure: 5.4%  Cancer: 10.2%  Chronic rejection: 8.4%  Respiratory failure: 11.7%  Other: 30.1%  Missing data: 10.6% | | |
| ***Single Center Studies in Europe*** | | | | | | |
| Hopital Beaujon, France (*Thabut, et al*) [33] | 1988 - 2001 | 28 | 12 | | Sepsis: 50.0%  Chronic rejection: 33.3%  Carcinoma: 8.3%  Primary graft failure: 8.3% | |
| University of Munich, Germany (*Neurohr, et al*) [34] | 1997 - 2008 | 76 | 33 | | Technical complications/others: 9.1%  CMV: 3.0%  Non-CMV pulmonary infection: 18.2%  BOS ≥ stage 1: 42.4%  Graft failure: 27.3% | |
| ***Single Center Studies in North America*** | | | | | | |
| Two centers (Johns Hopkins  Hospital and the University of Pittsburgh Medical Center), US (*Schachna, et al*)[39] | 1989 - 2002 | 70 | 23 | | | Primary graft failure and infection were the leading causes of death |
| University of California, Los Angeles, US (*Saggar, et al*) [42] | 2003 - 2007 | 38 | 11 | | | BOS complications: 27.3%  Sepsis/pneumonia: 36.4%  Community acquired adenoviral infection: 9.1%  Perforated bowel: 9.1%  Air embolus: 9.1%  Thorocosternotomy wound dehiscence: 9.1% |
| University of Alabama, US (*Wille, et al*) [45] | 1994 - 2004 | 48 | 24 | | | Infection: 25.0%  BOS: 20.8%  Primary grafit failure: 16.7%  Mulitple orgain failure: 12.5%  Airway dehiscence: 8.3%  Malignancy: 4.2% |
| Toronto Lung Transplant Group, Canada (*Grossman, et al*) [47] | 1983 - 1989 | 16 | 6 | | | Perioperative deaths: n=1 gram-negative sepsis; n=1 air embolism; n=1 viral pneumonia. Post-operative deaths, n=1 viral pneumonia; n=2 chronic rejection |
| Cleveland Clinic, US (*Mason, et al*) [48] | 1990 - 2005 | 82 | 40 | | | Respiratory infection 32.5%  Sepsis or multisystem organ failure: 22.5%  Malignancy: 12.5%  Other: 32.5% |
| Papworth hospital (UK) (*Meyers et al)[41]* | 1984 - 1994 | 11 | 6 | | | Death within 3 months: n=4 (67%) infection |
| Pulmonary Institute of Rabin Medical Center, Israel (*Rusanov et al)[69]* | 2009 | 20 | 3 | | | Acute lung rejection: 66.7%  Sepsis: 33.3% |
| Pulmonary Institute of Rabin Medical Center, Israel (*Shirit et al)[68]* | 2004 - 2005 | 15 | 3 | | | 2 of acute lung rejection and 1 of sepsis. |
| Mayo Clinic  Jacksonville Lung Transplant Database (*Erasmus et al)[70]* | 2001 - 20007 | 19^a^ | 5 | | | Graft failure: 40.0%  Lymphoma: 20.0%  Non small cell lung carcinoma: 20.0%  Sarcoma pleura: 20.0% |

^a^ IPF patients with large airway stenosis, and/or bronchomalacia
