# Supplementary material for: Lung transplantation in idiopathic pulmonary fibrosis: a systematic review of the literature
Source: BMC Pulm Med. 2014 Aug 16;14:139. doi: 10.1186/1471-2466-14-139 (PMC4151866; doi:10.1186/1471-2466-14-139)
Supplement: Additional file 4 — Post-transplant length of stay and adjunct interventions in IPF patients. [file 1471-2466-14-139-S4.doc]

**Additional File 4. Post-transplant length of stay and adjunct interventions in IPF patients**

|  | ***Days in hospital following transplantation*** | | |
| --- | --- | --- | --- |
| Davis, et al[46] | All adult IPF single-lung transplant recipients, Washington University/Barnes Hospital 1988 - 1993 | 16 | 37.2 ± 35.1  (ICU stay: 12 ± 19.9) |
| De Oliveira, et al[49] | Consecutive IPF lung transplant recipients, University of Wisconsin Hospital and Clinics, 1993 - 2009 | 79 | Pre-LAS: 23  Post-LAS: 11 (p<0.01)  Pre-LAS: ICU stay 6  Post-LAS: ICU stay 3 (p<0.01) |
| Meyers et al, [41] | All IPF lung transplant recipients, Washington University/Barnes Hospital, 1988–1998 | 45 | 22 (14–33)  ICU stay: 5 (4–12) |
| Teo, et al[28] | Adults with IPF (ICD-9 code) as primary inpatient diagnosis and inpatient lung transplantation codes, Nationwide Inpatient Sample database,1988–2006 | 231 | Length of stay ≥14 days: 116 (50.2%);  There was a non-significant trend for shorter length of stay in SLT vs BLT (46.2% LOS ≥14 days vs 58.7%, p=0.075). |
| Thabut, et al[33] | IPF lung transplant recipients, Beaujon Hospital, Clichy, France, 1988–2001 | 28 | longer median hospital stay (14 days [IQR, 9 to 24 days] vs. 17 days [IQR, 11 to 31 days]; P< 0.001) |
|  | ***Indicators of Inadequate Pulmonary Status*** | | |
| Davis, et al[46] | All adult IPF single-lung transplant recipients, Washington University/Barnes Hospital 1988 - 1993 | 16 | 25% needed tracheostomy (after ≥7 days on ventilator) |
| De Oliveira, et al[49] | Consecutive IPF lung transplant recipients, University of Wisconsin Hospital and Clinics, 1993 - 2009 | 79 | Inhalation of nitric oxide: 45 (57.0%)  ≥48 hours of nitric oxide inhalation: 17 (21.5%)  Reintubation: 16 (20.3%)  ≥48 hours of ventilator support: 31 (39.2%)  There were significant differences between Pre-LAS and LAS IPF patients in terms of postoperative inhalation of nitric oxide (36.4% vs 71.7%, p<0.01), 48+ hours of inhalation (33.3% vs 13.0%, p=0.03), length of mechanical ventilation (2.8 days vs 1.1 days, p<0.01), 48+ hours of ventilation (57.6% vs 26.1%, p<0.01), |
| Meyers et al, [41] | All IPF lung transplant recipients, Washington University/Barnes Hospital, 1988–1998 | 45 | Mechanical ventilation mean: 3 days (2–10 days)  Tracheostomy: 7/45 (15.6%)  There were no significant differences between SLT and BLT in terms of tracheostomy, or days of postoperative hospital stay, mechanical ventilation, or ICU stay. |
